# Pop-Cola Acids and Tooth Erosion: An *In Vitro*, *In Vivo*, Electron-Microscopic, and Clinical Report

**DOI:** 10.1155/2010/957842

**Published:** 2010-12-02

**Authors:** Amirfirooz Borjian, Claudia C. F. Ferrari, Antoni Anouf, Louis Z. G. Touyz

**Affiliations:** McGill Faculty of Dentistry, Montreal, PQ, H3A 2B2, Canada

## Abstract

*Introduction*. Manufactured Colas are consumed universally as soft drinks. Evidence about the acid contents of Cola-beverages and its effects on teeth is rare. *Aim*. To assess (i) cola acidity and buffering capacity *in vitro*, (ii) tooth erosion after swishing with colas *in vivo* (iii) scanning electron *microscopic effects* on teeth of colas, and tooth-brush abrasion, and (iv) report a *clinical case* of erosion from cola consumption. *Materials and Methods*. (i) We measured six commercially available pop “Cola beverages”, pH, and buffering capacities using a pH-Mettler Automatic Titrator, with weak solution of Sodium Hydroxide (ii) two cohorts, one *with teeth*, the second *without teeth* rinsed with aliquots of Cola for 60 seconds. Swished cola samples tested for calcium and phosphorus contents using standardized chemical analytical methods (iii) enamel, dentine, and the enamel-cemental junction from unerupted extracted wisdom teeth were examined with a scanning electron microscope after exposure to colas, and tested for tooth-brush abrasion; (iv) a clinical case of pop cola erosion presentation, are all described. *Results*. Comparisons among pop colas tested *in vitro* reveal high acidity with very low pH. Buffering capacities in millilitres of 0.5 M NaOH needed to increase one pH unit, to pH 5.5 and pH 7 are reported. Rinsing *in vivo* with pop cola causes leeching of calcium from teeth; SEM shows dental erosion, and pop-cola consumption induces advanced dental erosion and facilitates abrasion. *Conclusions*. (i) Pop-Cola acid activity is below the critical pH 5.5 for tooth dissolution, with high buffering capacities countering neutralization effects of saliva; (ii) calcium is leeched out of teeth after rinsing with pop colas; (iii) SEM evidence explains why chronic exposure to acid pop colas causes dental frangibles; (iv) a *clinical case* of pop-cola erosion confirms this.

## 1. Introduction

Acidic foods are consumed globally [1, 2] but universally assumed to be innocent as to their effects on the mouth. Acidic food and beverages can affect natural teeth, and chronic exposure often leads to the development of dental frangibles (attrition, erosion, abrasion, and decay) [3]. Not only is acid derived directly from colas, but also after imbibing the cola, acid is produced from biofilm bacteria metabolizing fermentable sugars in the drinks [4]. Human saliva acts as a neutralizing and/or buffering solution on imbibed acid beverages [4, 5]. Intraoral pH (pH 6.8) decreases from, after drinking, an acidulated drink to below pH5 within 2 to 3 minutes [5]. Oral acidogenic bacterial action on fermentable carbohydrates (monosaccharides like glucose and fructose; disaccharides like maltose and sucrose) also aggravates the pH reduction to below pH4. Intraoral pH takes about 25 minutes to change the acid environment, as further stimulated saliva neutralises any residual acid [5]. The critical pH, at which hydroxyapatite dissolves, is pH5.5, and because teeth are composed of *calcium-deficient carbonated hydroxyapatite*, they are vulnerable to decalcification in acid media [6]. Tooth material in saliva as a saturated solution of calcium and phosphate will show dissolution or remineralisation, depending on incumbent acidity of the surrounding saliva. This is illustrated by the following formula:

(1) $${\text{Ca}}_{10}{\left({\text{PO}}_{4}\right)}_{6}\;{\left(\text{OH}\right)}_{2}⟷10\text{C}{\text{a}}_{}^{2+}+6\text{P}{{\text{O}}_{4}}^{3-}\;+2\text{O}{\text{H}}^{-}.$$

Should the pH fall (increased H^+^ concentration and accordingly acidity), the 6PO_4_^3−^ ions convert to H_2_PO_4_^2−^ or H_2_PO^4−^, and the OH^−^ ions are neutralized to water, driving the reaction to the right, that is, dissolution of calcium. If the H^+^ concentration diminishes (i.e., pH rises and acid activity falls), the reaction is driven to the left producing remineralization [5, 6]. Repeated exposure to acid drinks changes the intraoral pH to below the critical pH (pH5.5), when chemical dissolution of calcium from dental hydroxyapatite occurs. This sustained low pH, <pH5.5, allows mordant development of frangibles starting and deriving from decalcification. Besides pH and buffering, calcium, phosphate, and to a lesser extent fluoride are important when considering erosive attack from beverages [6]. Chronic frequency of imbibing, method of drinking, timing of consumption, or type and quantity of beverage are known to influence the realization of frangibles [3]. The formulae and contents of pop-Cola beverages are closely guarded industrial secrets, and sparse data about acidity is provided on their consumer packages. In light of knowing the critical pH for hydroxyapatite, and also that acid drinks require a lot of alkaline salivary flow to neutralize dietary acid, it is most important to know the pH of pop-Cola drinks, the strength of their acids, particularly their buffering capacities as to how much alkali is needed to change their pH. Various methods are used to assess ultramicroscopic effects of beverages on teeth; these include surface hardness measurements, surface profilometry, iodide permeability tests, chemical analysis of dissolved minerals, microradiography, confocal scanning microscopy, quantitative light-induced fluorescence, atomic force microscopy, element analysis of solid samples, nano-indentation, ultrasonic measurements, and scanning electron microscopy (SEM) with environmental SEM (ESEM) [7]. With ESEM, minimal sample preparation is required, is economical, and minimizes risks for artefacts. Also ESEM and SEM permit progressive assessment of the identical test-tooth material without carbon or metal coating of native surfaces [7]. Recently (2010), Variable Pressure Scanning Electron Microscope (VP-SEM) has refined this technique further [8].

**Aim**

This study aims to (i) assess pH, and buffering capacities of six “pop-cola beverages” (initial rise of one pH unit to the critical pH and to neutral pH); (ii) measure calcium and phosphorous content of cola after rinsing from mouths with (dentate) and without teeth (edentulous); (iii) assess scanning electron microscopic (VP-SEM) erosive effects on native dental hard tissues after exposure to pop colas; (iv) describe a clinical case of pop-cola erosion.

## 2. Methodology

### 2.1. pH and Buffering

 Six pop colas, namely, Pepsi, Diet Pepsi, Coca Cola, Diet Coca Cola, and Selection Cola and diet Selection Cola were analyzed. Standard aliquots of Cola liquid were tested for pH and buffering capacity (Batches of potable over-the-counter Colas were purchased by chance at local retail outlets). The acidity as pH and buffering capacity was measured with an automated METTLER DL25 Titrator (AT) [9]. Buffering capacities were assessed with 0.5 M NaOH solution and titrated to raise the measured cola pH one pH unit, then to pH 5.5, and to pH 7. Two fresh 60mls aliquots were collected directly from each of 12 cans and placed in separate polystyrene Falcon test tubes for measurements. Titration measures were repeated for each sample from different cans in the same batch. For each of the 6 Colas, 24 titration measures were done.

**Automatic Titrator (AT)**

**Figure 1:**
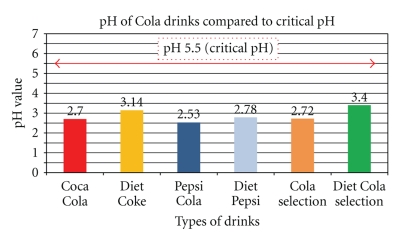
pH comparison among pop-Cola drinks. The pH levels of the six analyzed cola drinks are all significantly below the critical pH (pH 5.5) of Calcium hydroxyapatite (*P* < .001 Student-*t*). The diet colas are not necessarily more acidic; regular Pepsi Cola has the lowest pH (pH 2.53), while Diet Selection Cola is the highest pH (pH 3.40).

**Figure 2:**
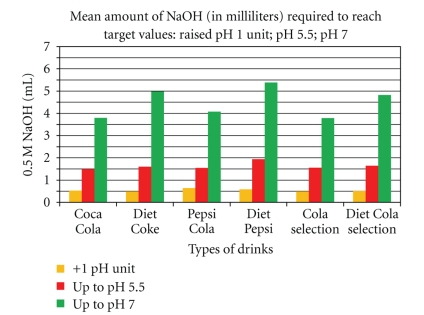
Buffering capacities of pop-Cola drinks for a single unit pH change (orange) to critical pH 5.5 (red) and pH 7 (Green). The orange bars show Pepsi Cola having the highest buffering capacity while Diet-Coke and Selection Cola share the lowest buffering capacity. The red bars show buffering capacities for change from the drink's initial pH up to the critical pH 5.5, with Diet Pepsi having the highest buffering capacity (1.9 mL of 0.5 M NaOH) while Coca Cola has the lowest buffering capacity (1.5 mL of 0.5 M NaOH). The green bars show buffering capacities for a pH change from the drink's initial pH up to neutral (pH 7.0). Diet Pepsi has the highest buffering capacity (5.38 mL of 0.5 M NaOH), regular Selection Cola (3.78 mL of 0.5 M NaOH) has the lowest buffering capacity, and Coca Cola (3.79 mL of 0.5 M NaOH) is very similar and not significantly different from Selection Cola. All the cola-drinks absorb the alkali but vary in the amount of alkali to reach pH 7.

The AT was used to determine the pH and buffering capacities of the various pop-cola liquids. For this analysis, the AT was used according to described methods by Larsen and Nyvad 1998 method [10] and measured initial pH's (Figure 1) and buffering capacities at the three different stated levels: (i) pH change of one unit from the incipient pH (orange bars, Figure 2), (ii) pH change up to the critical level pH 5.5 (red bars, Figure 2), and (iii) pH change up to the neutral level pH 7.0 (green bars, Figure 2) [11]. All measures were assessed at room temperature of 23°C. To ensure measurement accuracy and establish baseline controls, the AT was calibrated each time before use, at pH 2, 4, 7, and 10, using standard buffer solutions for all levels. A 60 mL volume of the tested sample was then titrated using a 0.5 M NaOH solution. Measures were repeated 24 times; the means of these are reported. See Appendix A.1.

### 2.2. Rinsing Pop Colas with and without Teeth

The six different pop-colas mentioned (Coca Cola, Pepsi Cola, Selection Cola, Diet Coke, Diet Pepsi, and Diet Selection Cola) were tested. Two volunteer cohorts, one fully dentate (mean age 22, M : F 4 : 2, *n* = 6) the second edentulous (mean age 52, M : F 3 : 3, *n* = 6), were used to swish with 20 mL aliquots of deionized Aquafina water as baseline and subsequently a 20 mL aliquots cola for 60 seconds for each volunteer. Every cola was sampled and analyzed twice, giving 12 analyses for swishes, using standardized analytical chemistry methods [12]. The colas from source and postswish expectorates were analyzed for calcium, and phosphorous contents using Inductively Coupled Plasma with Optical Emission Spectroscopy (ICP-OES) [12] and Ion Chromatography (IC) were used to determine concentrations of anions (Fluoride) present in the six colas direct from their cans. (See Appendices A.2 and A.3) For our analysis, concentrations of calcium and the major ions of interest as phosphate and fluoride are reported here [13]. To eliminate inter- and intraoperator bias, data was analyzed “blindly” by third-party technicians unaware of the source of procured samples.

**Results**

**Figure 3:**
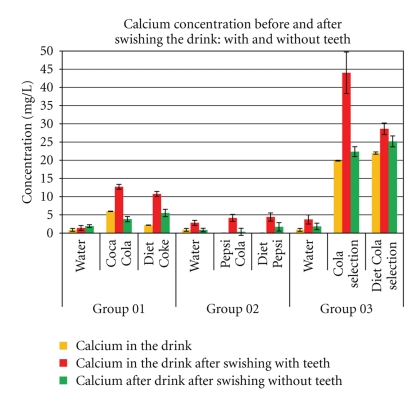
Calcium measures of swishes. Initial swishes were done for all groups with the same water (Aquafina) as controls. Subsequently, test standard-cola swishes were obtained; 2 hours later, diet equivalent swishes were secured. Calcium measures were done directly from source (bottled water or can) and the swished expectorates. Measures were assessed using ICP-OES. The Ca^2+^ content of control (water) remains constant for each group, but the Ca^2+^ content of swished water probes obtained from 3 separate cohorts (six volunteers for each group) vary slightly. Calcium was expressed as mg/L from the source and for test colas *after swishing with and without teeth*. There is a significant increase (*P* < .01 Student-*t*) in calcium, found in all the colas tested, when swishes with cola from subjects *with teeth* are compared to swishes of colas from subjects *without teeth*. The calcium content in the water controls is negligible.

Analysis reveals consistent significant (*P* < .01 Student-*t*) increases in calcium leeched from dentate subjects after swishing with all the pop-colas tested. The ICP-OES and IC measures were compared and there were no significant differences (*P* > .99) with regard to results obtained for phosphorous. Variable amounts of phosphorous were found in the various pop-colas tested, and this was reflected in the swish findings of phosphorous present after rinsing with these pop-colas. Hard tissue erosion, as chemical dissolution of calcium, may differ between colas, but is produced by all the colas tested. In none of the pop-colas is hard tissue erosive action absent from postswishing exposure, and *calcium content in the swish expectorates with teeth all showed significant *(*P* < .01* Student-t*)* increases in dissolved calcium when compared to swishing with water, or swishing without teeth* (See Figure 3).

### 2.3. Scanning Electron Microscopy

 Healthy teeth condemned for extraction (orthodontics or impactions) were used. Immediately after extraction, teeth were placed in deionized water refrigerated at 4°C for a maximum of two hours. Each cola was tested at three selected sites on the same tooth; this was repeated on three teeth from the same set of wisdom teeth, which provided fresh native material for assessment. For enamel, flat tooth surfaces were selected; for dentine, samples were obtained by cutting horizontally through the middle of the crown, and the enamel-cemental junction on each tooth used was also tested and examined directly. A Buehler saw with a diamond cutting blade was used to section the teeth [14]. After samples were cut, each specimen was rinsed with deionized water and air stream dried. All areas of focused interest were examined using scanning electron microscopy with VP-SEM [8]. Resolution was at 700X, with accelerating voltage 20–25 kV, to assess fracture patterns and morphology. Immediately after scanning controls, each sample was immersed in a cola for 30 s, and subsequently the specimens were immersed for a further 30 s (for a total of 60 s), and the *same location was* scanned and recorded. Unadulterated tooth surfaces of the same native precise areas were examined. No sputter, coating, metal or gold plasma enhancement was used. Image analysis of morphology, properties and characteristics (cracks, openings, and changes of identifiable shapes) were done with microscopic scales and by micrometer measures using the same microscope magnification of the identical areas tested. After 60 seconds of cola exposure, the specimens were brushed with a standardized rotary tooth brush for 5 seconds under 60 grams pressure. The specimens were scanned again after brushing.

**Results**

Ultrascaled measures (700X) reveal significant losses of hard tissue from erosion (*P* < .01, Student's *t*-test paired). Initial erosion is seen after exposure to the colas, as the chemical loss of calcium widens cracks and causes surface crenellations to develop. After 60 s cola exposure, soft surface crenellations develop. After brushing, evidence of tooth-brush abrasion is evident. See Figures 6–11.

Figures 6–11: VP-SEM images for each cola tested are presented. Enamel, ECJ and Dentin at 60 seconds exposure at ×700 show effects after brushing. Figure 6 is for Coca Cola at 700X. Figure 7 is for Pepsi Cola at 700X. Figure 8 is for Selection Cola at 700X.  Figure 9 is for Diet Coke at 700X. Figure 10 is for Diet Pepsi at 700X. Figure 11 is for Diet Selection Cola at 700X.

### 2.4. Clinical Case Report—See Figures 12(a)–12(j)

 An 18-year-old male presented complaining of temperature sensitive teeth and that his “teeth were getting smaller.” His medical history revealed nothing significant to indicate that he may suffer from GORD (Gastro-Oesophageal Reflux Disorder). The aetiology of GORD is variable, but when the gastric contents flow from the stomach to the mouth, it is called Gastro-oesophageal reflux disorder (GORD) [15]. Common symptoms are heart burn along the oesophageal pathway; epigastric pain localised over the stomach; regurgitation into the mouth; dysphagia with or without pain; noncardiac retrosternal pain; chronic coughing and sore throat from laryngitis; vocal hoarseness; a throat globus [16, 17]. The patient reported none of these signs or symptoms. He attended his general dentist regularly and brushed and flossed his teeth regularly, morning and evening every day. He used fluoridated toothpaste and used a new soft nylon tufted tooth brush every month. His diet was derived from foods in all food groups (meat/fish, dairy, grain/cereals, and vegetables), but he specifically stressed that he did not drink fresh fruit juices and rarely ate fresh fruit because “they hurt his teeth.” However, he reported drinking copious amounts of cola “solidly from my last two years of in primary school till now,” The time which was calculated to be about ten years. He drank all brands of conventionally (carbohydrate) sweetened colas, until three years prior to presenting, the point from which he changed to drinking exclusively synthetically sweetened “diet-colas.” A fortnight of dietary analysis confirmed these predilections and showed that he consumed between at least one litre to one and a half litres of cola daily, from cans, or bottles depending on availability. This daily cola intake was a habitual part of his diet, and he would “swish the cola over his teeth to reduce the gas and enhance the flavour.” 

 On presentation (Figure 12(a)–12(k)), his teeth appeared reduced in size apparently by erosion with reduced enamel and dentin exposure. The upper anteriors were more affected than the rest of the teeth, along with molar and premolar cuspal reduction and incisal attrition. The patient felt pain when gentle airstream was passed over his incisors. All the teeth reacted to thermal stimulation were vital. Staining of the teeth confirmed excellent oral hygiene (plaque index—modified O'Leary—below 5% of surfaces). Subgingival probing revealed no periodontal disease (no sulcus depth exceeding 3 mm), and further subgingival exploration showed only healthy tooth surfaces and no caries. 

## 3. Discussion

### 3.1. pH, Acidity, and Buffering

All the standard cola drinks show a progressive increased requirement of base to neutral pH 7, as does all the diet Cola beverages. The pH and buffering do not fully explain the erosive potential of colas, as the mineral content, concentrations of organic acids (phosphoric and citric), fluoride, and the ability of the mix to remove calcium from the mineral surface are all relevant [18]. However, pH-expressing acid content, buffering ability, and acidic ions available for the overall general mordant effect of acid-cola beverages are more important in producing erosion, as without an acid environment the other stated ions are not active. Figure 1 shows how low the pH of the selected group of pop Cola drinks is. All the cola drinks register pH values well below the critical pH 5.5, at which tooth decalcification occurs (Figure 1). Among the tested drinks, Pepsi Cola is the most acidic (pH 2.53) while Diet Selection Cola is the least (pH 3.4). From the results presented in Figure 2, Selection Cola and Diet Pepsi-Cola have the largest buffering capacities for a pH change of one unit. As for the pH change up to its critical value pH 5.5 (Figure 2), Pepsi Cola requires the largest amount of sodium hydroxide; Diet Pepsi Cola (Figure 2) finally appears to be the most resistant drink to a pH change up to neutralization (pH 7.0). 

These results are noteworthy as the low pH values reflect that the pop-Cola drinks are extremely acidic and consequently could all contribute to the decalcification of teeth (See Figures 6–11). It is also important to say that an average of 5.86 mL of base is required to bring the pH back to the neutral level of pH 7.0 (Figure 2). This indicates that not only are the cola drinks highly acidic when they are first exposed to teeth, but they would also require a large amount of alkaline-stimulated saliva to be neutralized.

### 3.2. Rinsing with and without Teeth In Vivo

**Figure 4:**
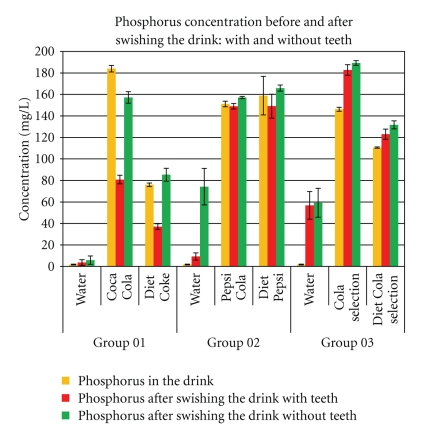
Phosphorous concentrations were expressed as mg/L from the source (bottled water or can) and swished test drinks. Aquafina water was used as control for all groups. Measures were assessed using ICP-OES. The phosphorus content of control (water) remains constant for each group, but the phosphorus content of swished water probes obtained from 3 separate cohorts (six volunteers for each group) vary. There is wide variation of phosphorous concentrations when swishes with cola from subjects *with teeth* are compared to swishes of cola from subjects *without teeth*. This is because there are variable amounts of phosphoric acid in the colas and phosphates in subjects' saliva; some reactive calcium-binding phosphorous is in stimulated saliva.

Whole saliva as a complex buffering solution may provide a definite protective buffering and neutralizing effect on acid dietary drinks [19–21]. But with acid drinks, including pop colas, this buffering/neutralizing effect may be overwhelmed [22]. The colas tested are not all chemically identical; yet they all leech calcium from teeth in vivo after contact for 60 seconds (Figure 3). The phosphorous content varies (Figure 4) because the phosphorous content of the colas from the cans varies. The dental and salivary calcium salts absorb some of the phosphorous from the cola; also the acidity from each cola varies as does the buffering, and reflex stimuli may vary in intensity of reaction. Other intraoral phosphate sources may derive from gingivo-crevicular fluid and calculus (tricalcium phosphate, octa-calcium phosphate, dicalcium diphosphate). Because the phosphorous composition of the colas is variable, the amount of phosphates produced after swishing is also variable (Figure 4). Although the chemical composition of the colas tested varied, they all had high buffering capacities and acid activity (pH) well below the critical pH 5.5. But even when accounting for any extra calcium released in stimulated saliva [16], the calcium found in swished expectorates from all these colas is consistently and significantly (*P* < .01 Student-*t*) higher when compared to the calcium content in the can. Tooth erosion, as chemical dissolution of calcium, is derived from an intraoral source and is reflected by an increased content of calcium in all the swishes from the colas tested. The increase in calcium shown in the swishing experiment *without teeth* (Figure 3) after rinsing with the colas may be derived from calcium ions secreted in stimulated saliva. Other sources of calcium may derive in miniscule aliquots from minor salivary glands and circulating oral glycoprotein. The calcium content in the expectorates varies, *but the only confounding factor, as a variable that explains the increase of calcium in the swish expectorates, is the presence of teeth*; this is particularly highlighted by comparisons in each cola of calcium content in the expectorate *swish with teeth* that shows significant (*P* < .01, Student-*t*) increases in dissolved calcium when compared to swishing with water, or *swishing without teeth.* Some phosphates may derive from stimulated saliva, and some from the teeth or other intraoral phosphate sources, but the amounts of calcium contents measured in the swishes cannot be explained in this experiment, from sources other than from the natural teeth *in vivo.*

**Figure 5:**
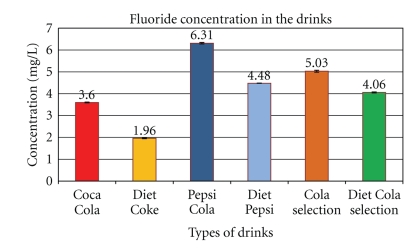
Fluoride concentration in pop colas with samples (*n* = 24) directly from the cans. The above graph shows the fluoride concentration (mg/L or ppm) in the six Cola drinks tested. Pepsi has the highest concentration (6.31 ppm) while Diet Coke has the lowest concentration (1.96 ppm).

Considering that the average rate of resting-saliva secretion is 0.78 mL/min, this would also mean that a long period of time is needed for the mouth to return to neutral pH 7 [4, 11]. Knowing that acids are the most potent stimuli for reflexive stimulated-salivary flow, the rate of stimulated secretion can increase to reach a maximum limit of 8ml saliva per minute [18]. Even if this maximum rate is reached when consuming cola drinks, neutralisation to physiological stable oral pH levels would still need a long period of time to be achieved, and the calcium content from saliva is too low to account for calcium increases after rinsing. Typically after one test bolus in the mouth this is about 25 to 30 minutes [4]. At 20 mg/L, F^−^ fails to reduce erosion of teeth *in vitro *[22], even with the mean fluoride content from the cans, it was 3.5 mg/L (ranging from 2 to 6 mg/L); this concentration does not stop calcium dissolution after swishing (see Figure 5). Increasing the fluoride content is feasible, but higher concentrations would be undesirable, as it would be unacceptably toxic and also negatively affect organoleptic taste properties. The dietary acids from the colas over power any protective effect from saliva and the same applies to the fluoride content of the colas. Some other important biological factors may slightly affect decalcification and tooth erosion, such as the saliva flow rate, its composition, buffering capacity and stimulation capacity, and the acquired pellicle, which has diffusion-limiting properties by its composition, maturation, and thickness [15]. The type of dental substrate and its density of composition also can effect erosion, as does the dental anatomy and occlusion influencing the flow of liquids over the tooth surfaces; and besides the anatomy and histology, the vigorous function of oral soft tissues in relationship to the teeth affects the development of erosion [15]. None of these are as important as the acid composition and pH of the pop-cola drink in producing erosion.

Keratosis on the tongue seems to act as a rasper that removes surface tooth material softened by decalcification. Decalcification caused by regurgitated gastric contents in bulimia often manifests first as palatal erosion because of tongue thrusting, removing softened tooth material [15, 23, 24]. The acidulated colas also act as a stimulus for stimulated saliva to flow which contains calcium. But the calcium content of saliva is negligible (mean 5.7 mg/100 mL; range: 2–10 mg/100 mL), and *even with maximum secretion rate of saliva with ranges *of 7 to 8 mL in one minute [25], comparisons between swish expectorates (*with and without teeth*) indicate that it is impossible for stimulated saliva to secrete calcium in amounts recorded after 60 seconds of swishing. Also it is inordinately difficult to procure age-matched (edentulous) controls without teeth below 35 (dentate controls with teeth had a mean age of 22 years old), or to find people aged 52 years (the mean age of the test edentulous group) without any dental restorative work. Consequently, age was discounted as a confounding factor in the comparisons.

### 3.3. Scanning Electron Microscopy

**Figure 6:**
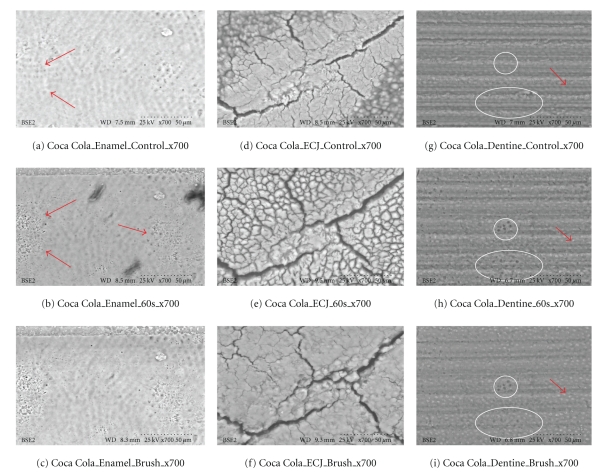
Coca Cola (×700). (a–c) The red arrows show developing erosive effects on smear layer over the surface of enamel; there is enamel erosion and minor abrasion as enamel is dense and hard. (d–f) show developing cracks and crevices over the ECJ surface which become aggravated due to loss of calcium. Also abrasion as loss of material after brushing, with the softened surface reflecting loss of detail going down to deeper more calcified layers, is seen in (f) compared to (e). Red arrow in (g) shows closed dentine tubules which are markedly opened in (h) after exposure to the cola. (i) shows loss of surface material, removed by abrasion, with some tubules (circled) becoming smaller, while others (red arrow) expose deeper levels of the tubes. These results correlate well with the calcium measured in expectorates from swishes with Coca cola in Figure 3.

**Figure 7:**
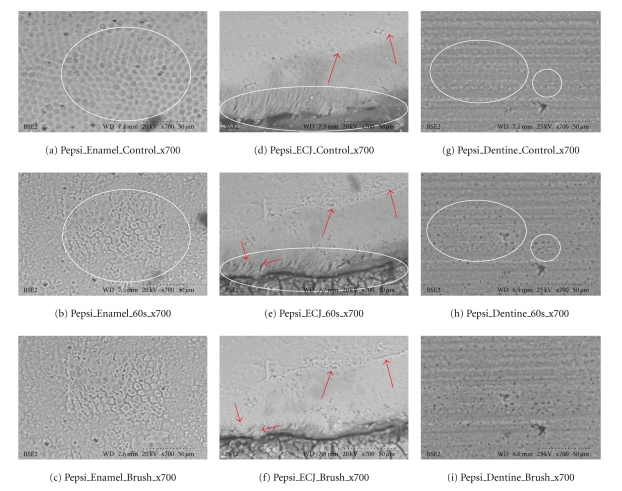
Coca Cola (×700). (a–i) White ovoid outlines show erosion in the same areas for enamel, ECJ, and dentine. Red arrows indicate obvious locations of change. There is minimal abrasion in (c), as enamel is dense, hard, and resistant to the brushing. But white outline in (e) shows erosion, and red arrows show loss of surface material from brush abrasion in (f). These SEM results are consistent with the calcium dissolution from swishes in Figure 3.

**Figure 8:**
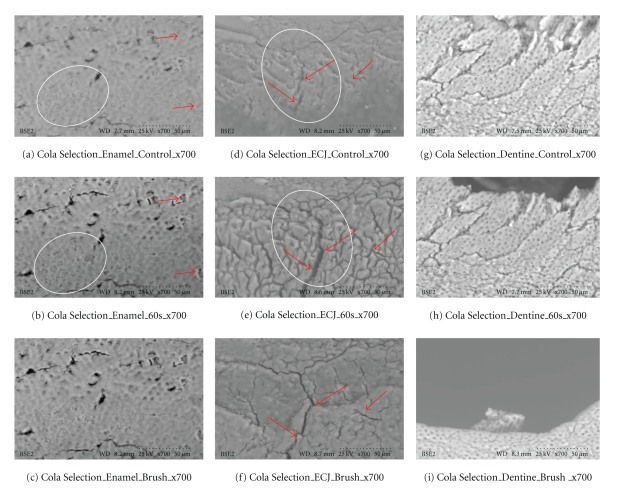
Coca Selection (×700). Outlined areas clearly show erosion with surface crenellations and profusion of shrinkage cracks from calcium loss when comparing (a), (d), and (g) to (b), (e), and (h). Minimal abrasion is present on the enamel, but red arrows from brush abrasion are clearly visible in (f); the surface cracks while enlarged from erosion appear narrower and less numerous, as the soft superficial material is lost to the action of the brush. These SEM results are also consistent with the calcium dissolution from swishes in Figure 3.

**Figure 9:**
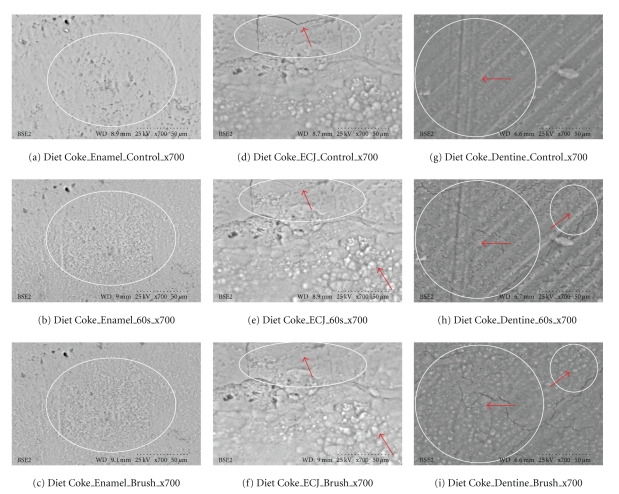
Diet Coke (×700). Outlined areas show erosion with minor surface changes from calcium loss when comparing (a), (d), and (g) to (b), (e), and (h). Minimal abrasionis is present on the enamel, but red arrows from brush abrasion are clearly (i). Red arrows show dentine tubules exposed from erosion appear clearer, wider, and more open as the soft surface material is lost to brush abrasion. These SEM results are also consistent with the calcium dissolution from swishes in Figure 3.

**Figure 10:**
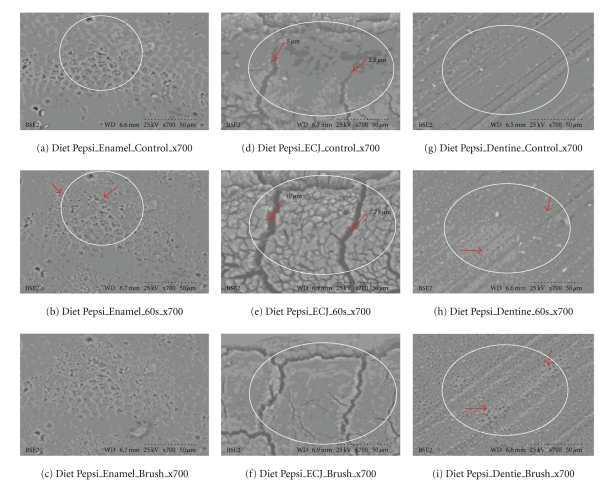
Diet Pepsi (×700). Outlined areas clearly show erosion with surface crenellations and profusion of shrinkage cracks from calcium loss when comparing (a), (d), and (g) to (b), (e), and (h). Minimal abrasion are present on the enamel, but red arrows from brush abrasion is clearly visible in (f); the surface cracks while enlarged from erosion appear narrower and less numerous, as the soft superficial material is lost to the action of the brush. Red arrows show that dentine tubules exposed from erosion appear clearer, wider, and more open as the soft surface material is lost to brush abrasion. These SEM results are also consistent with the calcium dissolution from swishes in Figure 3.

**Figure 11:**
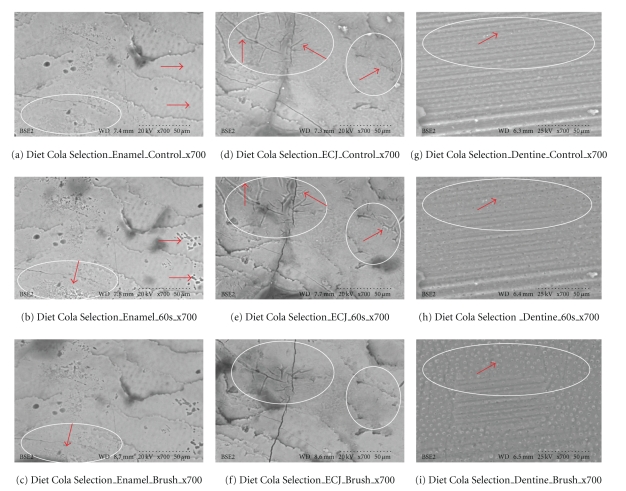
Diet Cola Selection (×700). White outlined areas clearly show erosion with surface crenellations and profusion of shrinkage cracks from calcium loss when comparing (a), (d), and (g) to (b), (e), and (h). Minimal abrasion is present on the enamel, but loss (red arrows) from brush abrasion is clearly visible in (f); the surface cracks while enlarged from erosion, appear narrower and less numerous, as the soft superficial material is lost to abrasion. Red arrows, (g)–(i), show that dentine tubules exposed from erosion appear clearer, wider, and more open as the soft surface material is lost to brush abrasion. These SEM results are also consistent with the calcium dissolution from swishes in Figure 3.

The recent increase of consumption of pop drinks [26] is reflected in increased reporting of dental erosion [3, 27]. Data reported here shows that damage occurs at a microscopic level and corroborates information gleaned from epidemiology and marketing [3]. Some consider a 30- or 60-second exposure unreasonably long; this is not valid criticism, as most reported data from other investigators uses target times in excess of 5 minutes, even hours or days of immersion [28], and 30 seconds and 60 seconds could well approximate the total time a 350 mL can of cola may be exposed to teeth, when people swallow a 60ml bolus and swish it for 5 to 10 seconds. Dental ravages from cola drinks have a high prevalence in children and young adults [7, 29]. Evidence presented here in this electron microscopic study re-enforces and confirms this theory. This study also demonstrates this interaction results in surface etching (Figure 6) and cracks (Figures 8 and 10). This SEM evidence confirms observations about smear layer removal, opening of dentine tubules, and increasing of tubules diameter based on randomly selected sites [30, 31]. Exposure of dentinal tubules by acidulated colas may result in dentine hypersensitivity [31]. This is probably due to disruption of fluid dynamics in the tubules as well as by mechanical loss of tooth material [30, 31]. The results also indicate that with light brushing after 60 s exposure to pop colas, abrasion will be obtained.

### 3.4. Clinical Case Report

**Figure 12:**
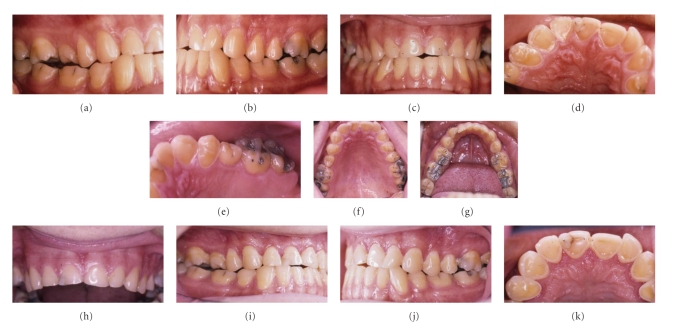
In (a–e), the teeth are smaller, with shiny surfaces. The full intercuspal occlusion shows spaces from reduction of cuspal height; the incisors, canines, and premolars are eroded palatally and worn down with attrition. (f, g) are occlusal views of Upper and Lower arches. Loss of occlusal tables from attrition and erosion is evident on the premolars and molars; there is occlusal saucerisation of cusps on the first and second molars. Figure 12(h) is a pre-op view of upper anteriors. Note short vertical height of incisors and loss of buccal enamel on all upper teeth. Figures (i, j, k) are healing post-op views after clinical crown lengthening. The central incisors are longer, the premolar palatal cusps are lost, and the palatal aspects show a clear palatal step where enamel has eroded away. This erosive pattern involves the buccal surfaces of the upper teeth and is decidedly different when compared to the erosive patterns encountered with GORD [17].

Tooth wear results for three main processes erosion, attrition, and abrasion, with chemical, physical, and physiological forces interacting to produce a clinical case of dental frangibles. The case presented shows all three major effects (Figure 12). Saliva may moderate these frangibles through pellicle formation and remineralisation processes. These protective influences are overwhelmed by frequent drinking of pop colas and frangibles result. Restorative therapy for frangibles varies with the extent of damage. Therapy ranges from eschewing acid drinks, avoiding brushing immediately after drinking pop-drinks, reducing frequency of drinking to fissure sealants and coating, occlusal build-up with overlays, or comprehensive oral rehabilitation with full-coverage crowns [32].

## 4. Concluding Remarks

(i) Pop-Cola drinks tested are acid and may decalcify tooth material. (ii)This study provides evidence that all these six common colas (Pepsi Cola, Diet Pepsi Cola, Coca Cola, Diet Coke, Selection Cola and Diet Selection Cola), leech calcium out of teeth, after rinsing with pop-colas. (iii) This SEM experiment shows that acid pop-cola ignores the smear layer, softens and erodes dental hard tissues, and facilitates abrasion. (iv) The clinical case report shows erosion from chronic imbibing of acid pop colas. These data collectively provide more evidence as a proof that chemical dissolution by tooth decalcification is caused by drinking pop colas. This study demonstrates clear visual evidence of dental erosion with altered enamel, and dentine morphology changes due to short exposure to pop colas. 

## Figures and Tables

**Figure 13:**
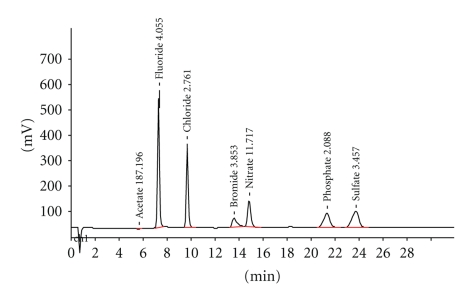
IC output for multi-standard solution. The concentration of the different ions can be obtained from the ion chromatograph.

## Appendix

### A. Descriptions for Technique Details of ICP-OES and IC

#### A.1. pH Measurement

The Automatic Titrator Instrument was standardized with certified reference buffers at pH 2, 4, 7, and 10. A combination electrode was used to measure both the reference materials and the colas. All measurements were completed at a measured temperature of 23°C. Each sample was measured 12 times.

#### A.2. Inductively Coupled Plasma-Optical Emittance Spectroscopy (ICP-OES)

For the purpose of our analysis, the ICP-OES was used to detect the concentrations of calcium, phosphorus (and sodium, potassium, boron, tin, arsenic, iron, nickel, lead, copper, magnesium, zinc, sulphur, chrome, cobalt, and manganese…not reported) present in the cola drinks being tested. In order to obtain accurate and precise results, twelve replicates for every drink being tested along with a deionized Aquafina water blank were prepared with due diligence. Initial Precleaned Polypropylene *Digi*TUBEs (50 mL) were clearly labeled, and 20 mL samples were dispensed to each tube with a pipette. Because there is a high sugar content in the regular drinks, a matrix effect is formed, preventing the ICP-OES from determining accurate concentrations of those elements being tested. To eliminate any potential matrix, 5 mL of Trace Metal Grade Nitric Acid was added to each sample, and the samples were digested to 95°C for 120 minutes using a 24-position *Digi*PREP equipped with a* Digi*PROBE. The probe was placed in a sample in order to maintain and monitor the digestion procedure. Once digestion was finished (1) the samples were cooled to room temperature, (2) volumes of twelve replicates and a blank were completed to 50 mL with the deionized water.

The same procedure was performed for each of the six cola drinks being tested. A calibration curve was prepared from 0.5 to 50 mg/L with certified ICP-OES standards diluted in a solution of 2% Nitric Acid. A total of four points including a calibration blank (consisting of 2% Trace Metal Grade Nitric Acid) were prepared. Furthermore, two Quality Control Standards were prepared from a different lot of certified ICP-OES standards. They were prepared at a concentration of 5 and 25 mg/L.

#### A.3. Ion Chromatography (IC)

The IC is used to determine the concentration of different anions present in the six cola drinks being tested. For the purpose of our analysis, the concentrations of the major anions, which are fluoride, acetate, formate, chloride, phosphate, nitrate, and sulphate were studied. Other ions were also assessed but not reported here.

A fixed volume (5 mL) of the cola is accurately delivered in a properly labeled *Digi*TUBEs (50 mL). The cola samples were then filtered using a very small filter (diameter of pores = 0.45 *μ*m) to remove all sediments and impurities present that would cause microbial alterations. A volume of 4 mL of every filtered sample is then collected in a container using a well-rinsed polypropylene vial.

A calibration curve was prepared using certified IC standards for all analytes. The calibration curve covered a range from 0.01 to 10 mg/L for each anion. Furthermore, two quality control standards were used at concentration of 0.02 and 5 mg/L.

Figure 13 shows an output of an ion chromatograph for a multistandard solution. Each peak on the graph represents a different ion. The species in the solution can be identified by their elution time. The concentration of each ion can then be obtained by evaluating the area under each peak. The concentration of the anions in the cola drinks was determined by plotting calibration curves.

## References

[1] Bawa S (2005). The role of the consumption of beverages in the obesity epidemic. *Journal of the Royal Society for the Promotion of Health*.

[2] Sirimaharaj V, Brearley Messer L, Morgan MV (2002). Acidic diet and dental erosion among athletes. *Australian Dental Journal*.

[3] Touyz LZG, Mehio A (2006). Dental Ravages from acidulated sof drinks. *Journal of Aesthetic and Implant Dentistry*.

[4] Johansson A-K, Lingström P, Imfeld T, Birkhed D (2004). Influence of drinking method on tooth-surface pH in relation to dental erosion. *European Journal of Oral Sciences*.

[5] Dawes C (2003). What is the critical pH and why does a tooth dissolve in acid?. *Journal (Canadian Dental Association)*.

[6] Featherstone JD, Lussi A (2006). Understanding the chemistry of dental erosion. *Monographs in Oral Science*.

[7] Attin T (2006). Methods for assessment of dental erosion. *Monographs in Oral Science*.

[8] Hitachi S-3000N Variable Pressure-SEM(VP-SEM) (2009). *Facility Electron Microscope Research, SEM Laboratory*.

[9] (2009). *METTLER DL25 Titrator*.

[10] Larsen MJ, Nyvad B (1999). Enamel erosion by some soft drinks and orange juices relative to their pH, buffering effect and contents of calcium phosphate. *Caries Research*.

[11] Morton DG, Smith ME (2001). The digestive system, basic science and clinical conditions. *The Mouth, Salivary Glands and Oesophagus*.

[12] Association of Official Agricultural Chemists (AOAC) (2009). Modified method 984.27, ICP OES. Calcium.

[13] Association of Official Agricultural Chemists (AOAC) (2009). Method 944.08 determines total fluoride.

[14] Schindler B (2006). *IsoMet Low Speed Saw*.

[15] Bartlett D, Lussi A (2006). Intrinsic cause of erosion. *Dental Erosion*.

[16] Lussi A, Jaeggi T, Lussi A (2006). Erosion. From diagnosis to therapy. *Ch 7.1.1 Chemical Factors*.

[17] Dawes C, Kubieniec K (2004). The effects of prolonged gum chewing on salivary flow rate and composition. *Archives of Oral Biology*.

[18] Imfeld T (1977). Evaluation of the cariogenicity of confectionery by intra-oral wire-telemetry. *Schweizerische Monatsschrift für Zahnheilkunde*.

[19] Imfeld TN, Myers HM (1983). Intraoral pH telemetry in man-materials and methods. *Identification of Low Caries Risk Dietary Components*.

[20] Tahmassebi JF, Duggal MS, Malik-Kotru G, Curzon MEJ (2006). Soft drinks and dental health: a review of the current literature. *Journal of Dentistry*.

[21] Hara AT, Lussi A, Zero DT (2006). Biological factors. *Monographs in Oral Science*.

[22] Valena V, Young WG (2002). Dental erosion patterns from intrinsic acid regurgitation and vomiting. *Australian Dental Journal*.

[23] Jenkins GN (1978). The physiology and biochemistry of the mouth. *Saliva Composition of Saliva*.

[24] http://www.zenithinternational.com/.

[25] Jain P, Nihill P, Sobkowski J, Agustin MZ (2007). Commercial soft drinks: PH and in vitro dissolution of enamel. *General Dentistry*.

[26] West NX, Hughes JA, Addy M (2001). The effect of pH on the erosion of dentine and enamel by dietary acids in vitro. *Journal of Oral Rehabilitation*.

[27] Johansson A-K, Johansson A, Birkhed D (1997). Dental erosion associated with soft-drink consumption in young Saudi men. *Acta Odontologica Scandinavica*.

[28] Lussi A, Jaeggi T, Zero D (2004). The role of diet in the aetiology of dental erosion. *Caries Research*.

[29] Jensdottir T, Arnadottir IB, Thorsdottir I (2004). Relationship between dental erosion, soft drink consumption, and gastroesophageal reflux among Icelanders. *Clinical Oral Investigations*.

[30] Jaeggi T, Lussi A, Grüninger A (2006). Restorative therapy of erosion. *Dental Erosion. From Diagnosis to Therapy*.

[31] Chandra A, Moazzez R, Bartlett D, Anggiansah A, Owen WJ (2004). A review of the atypical manifestations of gastroesophageal reflux disease. *International Journal of Clinical Practice*.

[32] Touyz LZG, Anouf A, Borjian A, Ferrari C (2010). Dental erosion and GORD. *Journal of Esthetic Dentistry*.

